# Exploration of a Novel Circadian miRNA Pair Signature for Predicting Prognosis of Lung Adenocarcinoma

**DOI:** 10.3390/cancers14205106

**Published:** 2022-10-18

**Authors:** Zhengrong Yin, Jingjing Deng, Mei Zhou, Minglei Li, E Zhou, Jiatong Liu, Zhe Jia, Guanghai Yang, Yang Jin

**Affiliations:** 1 Department of Respiratory and Critical Care Medicine, Hubei Province Clinical Research Center for Major Respiratory Diseases, NHC Key Laboratory of Pulmonary Diseases, Union Hospital, Tongji Medical College, Huazhong University of Science and Technology, Wuhan 430022, China; 2 Hubei Province Engineering Research Center for Tumor-Targeted Biochemotherapy, MOE Key Laboratory of Biological Targeted Therapy, Union Hospital, Tongji Medical College, Huazhong University of Science and Technology, Wuhan 430022, China; 3 Department of Thoracic Surgery, Union Hospital, Tongji Medical College, Huazhong University of Science and Technology, Wuhan 430022, China

**Keywords:** lung adenocarcinoma, circadian rhythm, miRNA, pairs, prognosis

## Abstract

**Simple Summary:**

Identifying new prognostic markers can provide a reference for the treatment of lung adenocarcinoma (LUAD) to improve its prognosis. Circadian rhythm disturbances are closely linked to the initiation, progression and prognosis of lung cancer. We aimed to explore the value of circadian miRNA (cmiRNA) as a prognostic marker of LUAD. A prognostic signature comprising seven pairs of cmiRNAs was established, and it exhibited excellent predictive value for overall and progression-free survival. High-risk patients showed higher sensitivity to primary chemotherapy drugs and targeted medicine compared with low-risk patients. Overall, the novel circadian-related miRNA pair signature could provide a precise prognostic evaluation with the potential capacity to guide individualized treatment regimens for LUAD. The cmiRNA–Cgenes network and corresponding enrichment analysis might provide clues for studying the underlying circadian dysregulation mechanisms involved in the progression of LUAD in the future.

**Abstract:**

Lung adenocarcinoma (LUAD) is the primary histological subtype of lung cancer with a markedly heterogeneous prognosis. Therefore, there is an urgent need to identify optimal prognostic biomarkers. We aimed to explore the value of the circadian miRNA (cmiRNA) pair in predicting prognosis and guiding the treatment of LUAD. We first retrieved circadian genes (Cgenes) from the CGDB database, based on which cmiRNAs were predicted using the miRDB and mirDIP databases. The sequencing data of Cgenes and cmiRNAs were retrieved from TCGA and GEO databases. Two random cmiRNAs were matched to a single cmiRNA pair. Finally, univariate Cox proportional hazard analysis, LASSO regression, and multivariate Cox proportional hazard analysis were performed to develop a prognostic signature consisting of seven cmiRNA pairs. The signature exhibited good performance in predicting the overall and progression-free survival. Patients in the high-risk group also showed lower IC50 values for several common chemotherapy and targeted medicines. In addition, we constructed a cmiRNA–Cgenes network and performed a corresponding Gene Ontology and Gene Set enrichment analysis. In conclusion, the novel circadian-related miRNA pair signature could provide a precise prognostic evaluation with the potential capacity to guide individualized treatment regimens for LUAD.

## 1. Introduction

Lung cancer remains the leading cause of cancer-related deaths in the world [1,2], with lung adenocarcinoma (LUAD) being the primary histological subtype [3,4]. The prognosis of patients with LUAD is markedly heterogeneous [5]. Therefore, the identification of effective biomarkers that are related to prognosis and drug efficacy in patients with LUAD is important: (1) providing early intervention and assisting clinical strategies to improve prognosis; and (2) revealing the underlying mechanism that may contribute to the discovery of new underlying therapeutic targets for preventing lung adenocarcinoma recurrence or progression.

Chronic circadian rhythm disturbances are closely linked to the initiation, progression and prognosis of tumors [6,7]. For example, a long period (more than 20 years) of shift work is related to an increased risk of breast and prostate cancers [8]. Disturbed daily sleep–activity cycles often occur in patients with lung cancer [9]. Mice harboring key circadian rhythm gene alterations accelerate the initiation and progression of lung cancer [6,10]. A study by Ye et al. revealed extensive alterations in clock genes across multiple cancer types. The correlations between clock genes, key oncogenic pathways, and clinical features might explain the underlying mechanisms of circadian rhythm disruption and tumor progression [11]. In addition, metastatic colorectal cancer patients with circadian rhythm disruptions have significantly lower survival rates and poorer quality of life than those with normal circadian rhythms [7,12]. The rhythm of cortisol is relatively “flat” in some lung cancer patients with poor survival [13]. Therefore, circadian rhythm disturbance may serve as a potential prognostic risk factors for lung adenocarcinoma.

The microRNAs participate in crucial physiological balance and pathological processes, including the malignant characteristics of cancer, and have been identified as excellent signatures associated with the diagnosis and prognosis of cancer [14,15,16]. Increasing evidence indicates that miRNAs play a major role in circadian rhythm regulation [17,18,19]. However, the value of circadian miRNAs (cmiRNAs) in lung adenocarcinoma has not yet been investigated. Moreover, a gene pair-based strategy is a promising approach that is compatible with the data from various gene detection platforms and has a wide range of applicability [20]. Therefore, our study aimed to develop a personalized prognostic signature for patients with LUAD based on cmiRNA pairs. We also preliminarily explored the value of the signature in guiding treatment approaches and the potential mechanism.

## 2. Materials and Methods

### 2.1. Study Design and Patients

We downloaded the miRNA and RNA-seq expression data of LUAD from TCGA and GEO databases. Patients lacking corresponding clinical information and those who survived for <30 days were excluded. Postoperative tumor and adjacent normal tissues of 11 patients with LUAD in the Union Hospital (Wuhan, China, WHUH cohort) were collected. All the patients signed an informed consent form. The study complied with the Declaration of Helsinki and was approved by the Institutional Review Board of Union Hospital, Tongji Medical College, Huazhong University of Science and Technology (protocol: [2020](S363), 30 December 2020).

**Figure 1:**
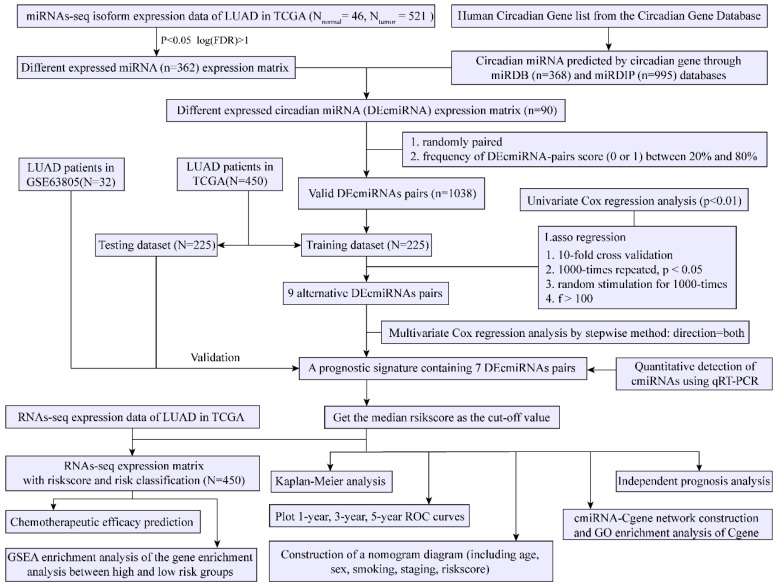
Overview of the study workflow.

The samples and data were collected from 10 March to 10 July 2022. Figure 1 shows the overall design of this study. The TCGA dataset was randomly split into training and testing datasets. The GSE63805 dataset was selected for independent validation.

### 2.2. Pairing of Differentially Expressed Circadian miRNAs (DEcmiRNA)

Circadian genes (Cgenes) were retrieved from the circadian gene database (CGDB, http://cgdb.biocuckoo.org (accessed on 5 March 2022)) [21]. Circadian miRNAs (cmiRNAs) were predicted according to the Cgenes through the microRNA data integration portal (miRDIP) database (http://ophid.utoronto.ca/mirDIP/ (accessed on 20 March 2022)) [22] and the microRNA target prediction (miRDB) database (http://www.mirdb.org (accessed on 21 March 2022)) [23]. To improve the accuracy of predictions, we set strict filter criteria as follows: mirDIP (score class: very high, integrated score more than 0.5 and number of sources more than 8); and miRDB (Include functional miRNAs only and exclude those with a target prediction score below 80, and those with more than 800 predicted targets in the genome). The “edgeR” package was employed in screening out the differentially expressed miRNAs (DEmiRNAs) between tumor and normal tissues (|log_2_FC| > 1 and FDR < 0.05). Then the differentially expressed cmiRNAs (DEcmiRNAs) were obtained via the intersection of DEmiRNA sets with cmiRNAs predicted by the mirDIP and miRDB databases for subsequent pairing and construction of the model.

This pairing strategy was consistent with that reported in previous studies [20,24]. Specifically, cmiRNAs were randomly paired. If the value of cmiRNA x was greater than that of cmiRNA y, then the score of cmiRNA pair (cmiRNA x/cmiRNA y) was defined as 1, and 0 otherwise. Moreover, cmiRNAs pairs with frequencies of 0 or 1 above 80% or below 20% were considered unrelated to prognosis and therefore excluded.

### 2.3. Establishment and Verification of a Prognostic cmiRNA Pair Signature

Clinical data (including survival information) were integrated into the cmiRNA pairing matrix. In the training dataset, prognostic cmiRNA pairs were filtrated by univariate Cox regression (*p* < 0.01). We further performed LASSO to minimize the risk of overfitting. Finally, the stepwise regression method (method = “both”) and the Akaike Information Criterion (AIC) were applied to screen cmiRNA pairs to construct the optimal Multivariate Cox regression model with the formula as follows: Risk score = h_0_ (t) × exp (score of cmiRNA pair 1 × β_1_ cmiRNA pair 1 + score of cmiRNA pair 2 × β_2_ cmiRNA pair 2 +……+ score of cmiRNA pair n × β_n_ cmiRNA pairs n). The risk scores of the samples in the testing and validation datasets were calculated on the basis of the above formula. The median risk score was set as the cutoff point for the high- or low-risk group classification.

Receiver operating characteristic (ROC) curve and Kaplan–Meier survival analysis were performed to evaluate the correlation between the signature and prognosis in the training, testing, total TCGA, and independent GEO (GSE63805) datasets. Moreover, we assessed the predictive value of the signature for progression-free survival (PFS) in the TCGA dataset. To further validate the clinical implications of the cmiRNA pair signature, we conducted multivariate analyses to explore the correlation between the signature and clinical features. The predictive values of the signature and the clinical variables for prognosis were also compared.

### 2.4. Formulation and Assessment of the Nomogram

Based on the results of multivariate analyses, we integrated smoking, age, sex, stage, and risk score into a composite nomogram by applying Cox proportional hazards regression to the TCGA dataset. The prognostic accuracy of the nomogram is shown by the ROC and calibration curves. The “M” stage (28% missing) was not included to ensure consistency. The R package “rms” was employed in drawing the nomogram plot.

### 2.5. Analysis of Tumor Immune Microenvironment

Immune cells in the tumor microenvironment were estimated using seven previously reported algorithms [25,26,27,28,29,30,31]. Spearman correlation analyses were conducted to investigate the correlation between the risk score and the immune cell composition. The Wilcoxon signed-rank test was performed to explore the differences in immune cells between the high- and low-risk patients.

### 2.6. Assessment of Drug Sensitivity

The IC50 values of common antitumor medicines for each sample were predicted using pRRophetic [32]. The Wilcoxon signed-rank test was applied to compare IC50 between high- and low-risk patients.

### 2.7. MiRNA Extraction and Quantification

Total RNA from postoperative tumor and normal tissues was extracted by RNAiso Plus (#9109 Takara Bio, Beijing, China). The RNA concentration was measured via NanoDrop 2000 (NanoDrop Technologies, Wilmington, DE, USA). The miRNA first-strand cDNA was synthesized using a tailing reaction (#B532451, Sangon Biotech, Shanghai, China) with total RNA (1 µg). Forward primers for the miRNAs (listed in Table S1) were obtained from Sangon Biotech. Quantitative real-time polymerase chain reaction (qRT-PCR) was carried out using the TB Green^®^ Premix Ex Taq™ II (#RR820A, Takara Bio, Beijing, China) and CFX96 Real-Time PCR Detection System (Bio-Rad, Hercules, CA, USA). The relative expression of miRNAs was calculated using the 2^−∆∆Ct^ method, with U6 as an internal reference.

### 2.8. Key cmiRNA–Cgene Network Construction and Gene Ontology (GO) Enrichment Analysis

The Cgenes matched to the cmiRNAs of the signature were obtained by the intersection of the above cmiRNA-target Cgene prediction analysis from the mirDIP and miRDB databases. Differential expression of the cmiRNAs and Cgenes in tumor and normal tissues in the TCGA cohort was calculated. Experimentally confirmed cmiRNA-target Cgene relationships were retrieved from the miRWalk database for miRNA-target interactions (which includes data from the miRTarBase database) [33]. We then used Cytoscape software to construct the cmiRNA–Cgene network. Spearman correlation analysis between cmiRNA expression and target Cgene in the TCGA-LUAD cohort was conducted using the Origin 2021 software. GO enrichment analysis of these target Cgenes was conducted using the DAVID database (https://david.ncifcrf.gov (accessed on 18 April 2022)).

### 2.9. Gene Set Enrichment Analysis (GSEA)

GSEA was performed using GSEA v4.2.1 software to explore the enrichment pathways and biological processes between the high- and low-risk groups in the TCGA dataset. Cancer hallmark and circadian rhythm-related gene sets were also investigated.

### 2.10. Statistical Analysis

Data analysis and graphical presentation were performed using R (version 4.1.3), Perl (version 5.32.1.1), SPSS Statistics 26, Adobe Illustrator and Origin 2021 software. Statistical significance was set at *p* < 0.05 (unless otherwise specified).

## 3. Results

### 3.1. Construction of the Prognostic Signature Based on the cmiRNAs Pairs

**Figure 2:**
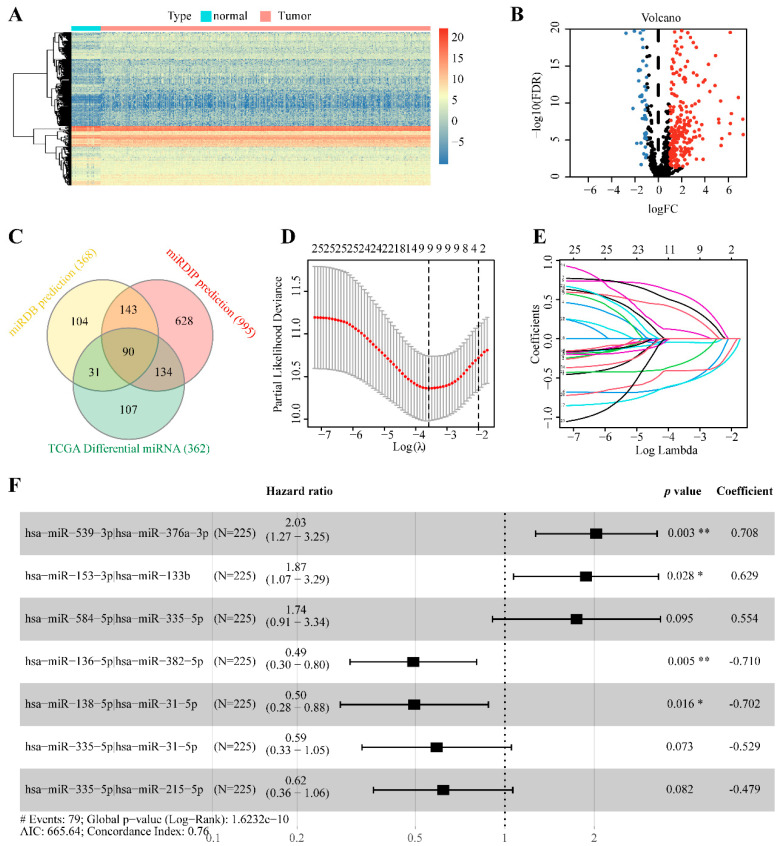
Construction of a prognostic model using cmiRNA Pairs. (**A**) Visualization of miRNA expression and (**B**) differentially expressed miRNA between tumor and normal tissues (red and blue dots indicate up-regulated and down-regulated miRNAs, respectively). (**C**) Venn diagram showing the number of differentially expressed circadian miRNAs (DEcmiRNAs). (**D**,**E**) Filtration of the DEcmiRNAs pairs using LASSO regression analysis (each differently colored line represents a candidate variable in the LASSO regression analysis). (**F**) The forest map showed the seven cmiRNA pairs of the established prognostic signature. (* *p* < 0.05; ** *p* < 0.01).

The flow diagram of the study is shown in Figure 1. First, we retrieved the miRNA-seq data of 46 normal and 521 tumor samples from the TCGA-LUAD cohort and a list of the circadian genes from the CGDB database (Table S2). A total of 368 and 995 cmiRNAs were predicted based on the circadian genes using miRDB and miRDIP databases, respectively (Table S3). A total of 362 DEmiRNAs between normal and tumor samples were screened out (Figure 2A,B). A total of 90 alternative DEcmiRNAs were obtained by the intersection of cmiRNAs (predicted by miRDB and miRDIP databases) and DEmiRNAs (Figure 2C). Then, 1038 valid cmiRNA pairs were identified and filtered after random pairing of the 90 DEcmiRNAs.

450 LUAD patients with complete clinical data and survival time > 30 days in the TCGA dataset were randomly divided into a training dataset (225 cases) and a testing dataset (225 cases). The clinicopathological features of patients in each dataset are shown in Table 1. In the training dataset, 26 survival-related cmiRNAs pairs were extracted using univariate Cox regression analysis (*p* < 0.01, Table S4). Then, nine cmiRNA pairs were screened out by LASSO regression analysis to prevent overfitting (Figure 2D,E). Finally, seven cmiRNA pairs were selected using the stepwise regression method (method = “both”) based on AIC to construct a prognostic signature using the multivariate Cox proportional hazard analysis (Figure 2F).

### 3.2. Evaluation and Validation of the Prognostic Signature

**Figure 3:**
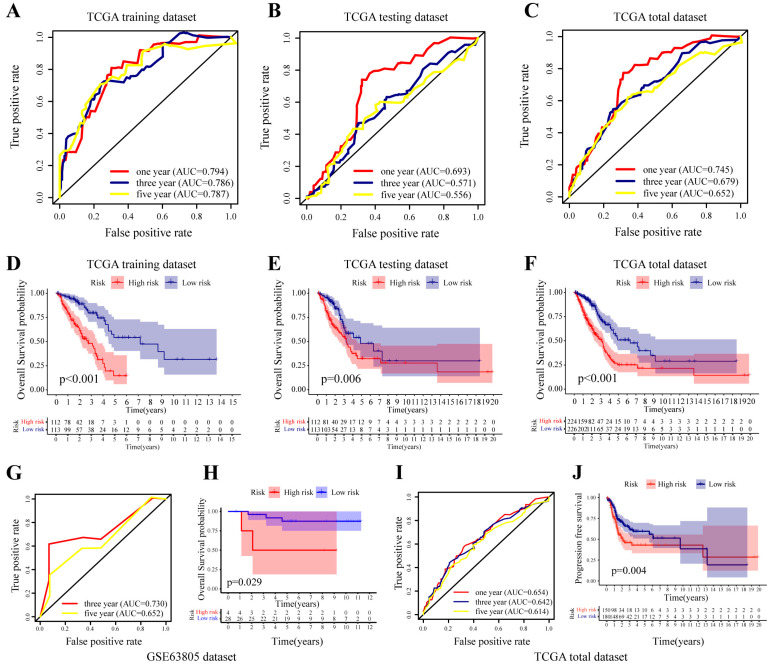
Evaluation and validation of the prognostic model. Receiver operating characteristic (ROC) curves for overall survival (OS) of (**A**) TCGA training dataset, (**B**) TCGA testing dataset, (**C**) TCGA total dataset, (**G**) GSE63805 dataset. Kaplan–Meier OS analysis of (**D**) TCGA training dataset, (**E**) TCGA testing dataset, (**F**) TCGA total dataset, (**H**) GSE63805 dataset. (**I**) ROC curves for progression-free survival (PFS) of TCGA total dataset. (**J**) Kaplan–Meier PFS analysis of TCGA total dataset.

The testing dataset and GSE63805 cohort were used for internal and external independent verification of the prognostic signature, respectively. Clinicopathological data and the corresponding miRNAs in the GSE63805 cohort are presented in Table 1 and Table S5, respectively. The risk scores of the cases in the testing and GSE63805 datasets were calculated using the prognostic model. The ROC curves of the signature for 1-, 3-, and 5-year overall survival in the TCGA training dataset, TCGA testing dataset, TCGA total dataset, and GSE63805 dataset were drawn (Figure 3A–C,G). The AUC values for the training, testing and independent GSE63805 dataset were 0.794, 0.693 and 0.730, respectively. We determined the median risk score of the training dataset as the critical value for high- or low-risk classification. Kaplan–Meier curves indicated that the low-risk patients had significantly longer overall survival than the high-risk patients across all datasets (*p* < 0.001) (Figure 3D–F,H). Additionally, the risk score model also showed a certain capacity to predict 1-, 2-, and 3-year PFS, and low-risk patients had longer PFSs (Figure 3I,J). All of the above results showed the robust prognostic power of the established signature.

**Figure 4:**
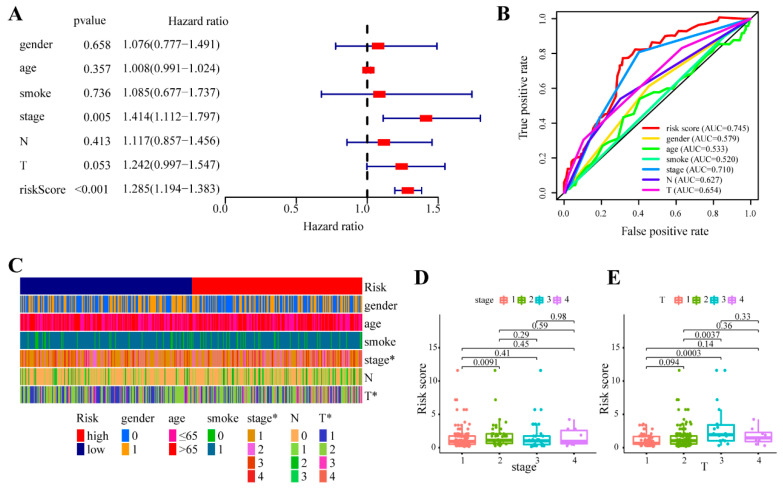
Relationship between the risk score and clinical variables. (**A**) The forest map shows the risk score and of the clinical variables. (**B**) ROC curves of the risk score and clinical variables for overall survival in the TCGA total dataset. (**C**) The heatmap of the clinicopathological data of patients between the low- and high-risk groups. The boxplots depict the relationship between the risk score and (**D**) clinical stage and (**E**) T stage. (* *p* < 0.05).

We further explored the association between the risk score and clinicopathological features. Multivariate Cox regression analysis indicated that clinical stage (*p* < 0.001, hazard ratio [HR] = 1.414, 95% confidence interval [CI] [1.112–1.797]) and risk score (*p* < 0.001, HR = 1.285, 95% CI [1.194–1.383]) remained independent prognostic factors after adjusting for clinical and pathologic factors (Figure 4A). The risk score indicated the optimal AUC value compared with sex, age, smoking, clinical stage, T and N stage (Figure 4B). Strip illustration (Figure 4C) and scatter drawings (Figure 4D,E) showed that there were significant correlations between the risk score and clinical stage and T stage.

### 3.3. Integrated Nomogram Combining the Risk Score with Clinical Variables

**Figure 5:**
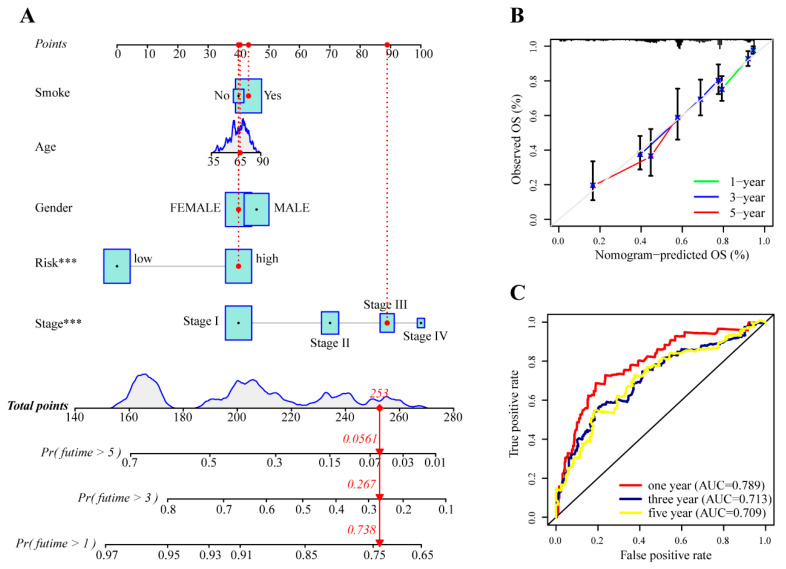
Integrated nomogram combining the risk score with clinical variables. (**A**) The visualization of overall survival prediction with risk score and clinical variables in the nomogram (*** *p* < 0.001). (**B**) The calibration curves of the nomogram. (**C**) ROC curves of the nomogram.

To further improve accuracy and practicality, we combined significant clinical factors (smoking, age, sex, and clinical stage) and risk score to fit a Cox proportional hazards regression model using the total TCGA dataset (Figure 5A). As shown in the nomogram, each factor was assigned a point and the total nomogram point was calculated from the sum of the individual points of all predictors. In association with the total points, the survival rate of patients can be estimated by projecting the total points downward. The calibration (Figure 5B) and ROC curves (Figure 5C) indicated that the integrated nomogram achieved a higher accuracy of survival estimation than the risk score alone.

### 3.4. Correlation between Risk Score and Tumor Immune Microenvironment

The immunosuppressive microenvironment is one of the main factors contributing to poor prognosis in patients with LUAD. Therefore, we tested the correlation between the risk score and the tumor immune landscape (Figure S1A–I). The results indicated that the risk score had a negative correlation with the majority of immune cells, especially crucial anti-tumor immune cells (such as CD8 + T and NKT cells), but had a positive relationship with tumor-promoting Th2 cells in the tumor microenvironment.

### 3.5. Application of Risk Score in Predicting Primary Drug Efficacy

**Figure 6:**
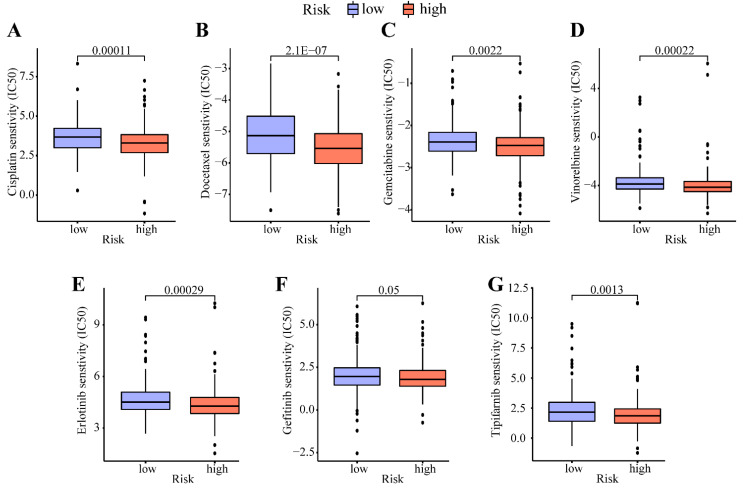
Association between the risk score and drug susceptibility. The boxplots show the difference in the half maximal inhibitory concentration (IC50) of (**A**) cisplatin, (**B**) docetaxel, (**C**) gefitinib, (**D**) vinorelbine, (**E**) erlotinib, (**F**) gefitinib, and (**G**) tipifarnib between the high- and low-risk group.

Based on the pRRophetic algorithm, we explored the correlation between the risk score and drug sensitivity in the TCGA dataset. As shown in Figure 6, high-risk patients had lower IC50 values than low-risk patients for several common chemotherapy drugs, indicating that the former were more susceptible to cisplatin, docetaxel, gemcitabine, and vinorelbine (Figure 6A–D). Similar results were observed for targeted therapy drugs, including the tyrosine kinase inhibitors erlotinib and gefitinib and the farnesyltransferase inhibitor tipifarnib (Figure 6E,F). The above results indicate the potential of this signature to predict drug sensitivity and guide treatment.

### 3.6. cmiRNA Quantitative Verification and Key cmiRNA—Cgene Network Construction

**Figure 7:**
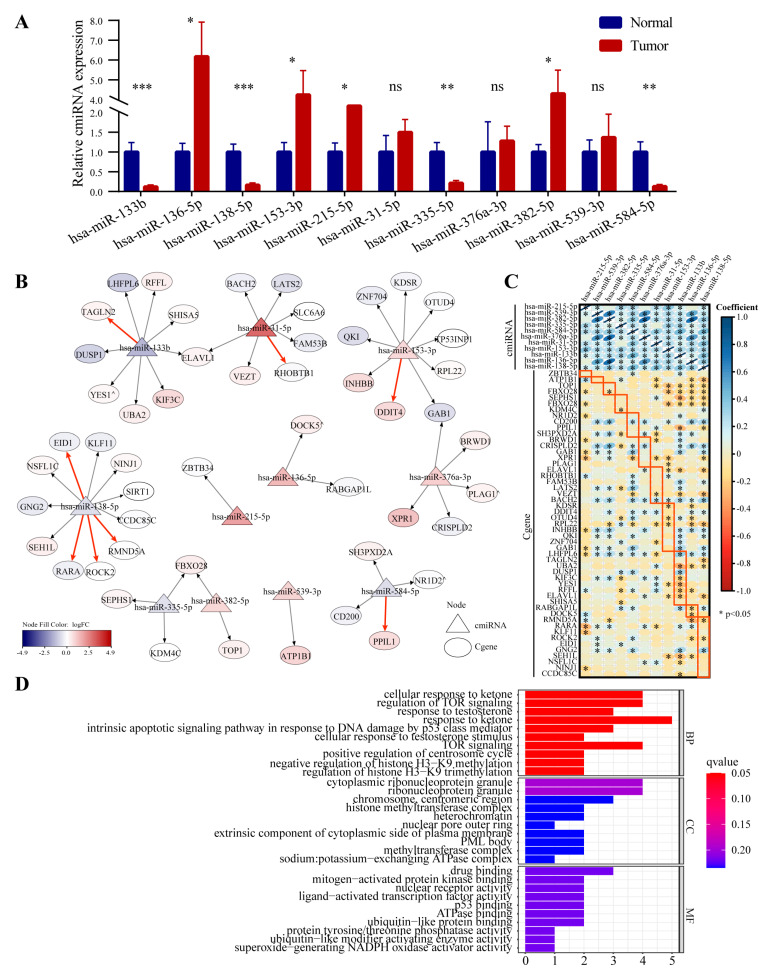
cmiRNA–Cgene network analysis and Gene Ontology (GO) enrichment analysis. (**A**) cmiRNAs of the signature quantification using qRT-PCR in tumor and normal tissues of 11 LUAD patient. (**B**) cmiRNAs and targeted-Cgenes network analysis (blue, white, and red mean lower, equal and higher expression level of cmiRNAs/Cgenes in tumor comparing to normal tissues, respectively, all *p* < 0.05 except that ^ *p* > 0.05, the red arrows indicate experimentally validated targeting regulatory relationships retrieved from the miRWalk database). (**C**) Spearman correlation analysis between miRNA expression and target genes in TCGA-LUAD cohort, the red box marks the target Cgene corresponding to each column of cmiRNA (**D**) GO enrichment analysis of the targeted Cgenes. (ns, not significant; * *p* < 0.05; ** *p* < 0.01; *** *p* < 0.001).

The relative expression of the 11 cmiRNAs between cancerous and adjacent normal tissues of patients with LUAD were further quantified by qRT-PCR. Only eight out of 11 miRNAs were confirmed to be significantly different between tumor and normal tissues, which might be explained by the low number of samples. (Figure 7A). The 48 targeted Cgenes matched to the 11 cmiRNA were extracted, and the key cmiRNA–Cgene network was established (Figure 7B). The relative expression levels (tumor vs. normal tissues) of cmiRNAs and their target Cgenes are also displayed in Figure 7B and Table S6. In addition, we searched the miRWalk database for experimentally validated target genes of these miRNAs (sourced from the miRTarBase database), which are marked by the red arrow in Figure 7B and listed in Table S7. It is clear that most relationships between these cmiRNAs and target Cgenes require experimental verification. Therefore, we performed Spearman correlation analysis between these cmiRNA expression and target Cgenes in the TCGA-LUAD cohort (Figure 7C). The expression correlation between Mir-138-5p and the ROCK2 and RMND5A genes was consistent with the classical negative regulation relationship between miRNA and target gene expression. GO enrichment analysis suggested that these targeted Cgenes were enriched in pathways including TOR signaling, response to DNA damage by P53 class mediator, and regulation of centrosome cycle (Figure 7D). GSEA between the high-risk and low-risk groups showed that most of the HALLMARK pathways, circadian regulation of gene expression, and cell cycle-related signaling pathways were active in high-risk patients (Figure S2A–C). Overall, these results might provide clues to explore new potential mechanisms of cmiRNAs and Cgenes that promote lung cancer progression.

## 4. Discussion

The five-year survival of lung cancer is 10–20% in most countries [34]. It has increased in some countries over the past 20 years due to early diagnosis, improved treatment methods, and the development of precision medicine. Prognostic biomarkers for the risk stratification of patients with lung cancer and providing references for clinical interventions are an important part of precision medicine. Chronic circadian rhythm disturbances are closely linked to the initiation and progression of tumors [6]. Some studies have shown that circadian genes have promising potential as prognostic markers of lung cancer. The miRNAs have unique advantages as prognostic markers. In this study, we established a signature, consisting of seven cmiRNA pairs, to predict the prognosis of lung cancer for the first time. The prognostic model has good predictive performance for the overall and progression-free survival of patients with LUAD in the TCGA dataset, and it has been validated in the GEO dataset (external validation). In addition, it has important reference significance in guiding the treatment of patients with LUAD. Nomograms also increase the usability and readability of the results.

Studies have suggested that clock genes and other genes related to the killing function (e.g., cytolytic factors, perforin, and granzyme B) have a circadian oscillatory rhythm, which is altered by chronic shift-lag. These alterations may be associated with the inhibited circadian rhythm of the cytolytic activity of NK cells and the progression of lung cancer [35]. Another study showed that lung cancer can induce deep reprogramming of the liver circadian rhythm at both the transcript and metabolite levels [36]. Further studies on the mechanism of circadian rhythm disorders leading to poor prognosis of lung cancer can provide potential therapeutic targets. The role of all 11 cmiRNAs in our signature in NSCLS has been studied to varying degrees. MiR-539-3p [37], miR-584-5p [38], miR-138-5p [39], miR-335-5p [40], miR-376a-3p [41], MiR-133b [42], MiR-382-5p [43], and miR-215 [44] were suggested to suppress the proliferation, migration, and invasion of NSCLC. While miR-153-3p [45], and miR-31-5p [46] were shown to promote the metastasis and invasion of lung cancer cells. In addition, studies have shown that miR-539-3p [47] and miR -138-5p [48] enhances chemosensitivity to cisplatin in NSCLC, and miR-136-5p promoted anlotinib resistance in NSCLC [49]. These studies suggest that these miRNAs play important roles in cancer development. However, whether they are involved in circadian rhythm regulation and how they contribute to the progression of lung cancer requires further study.

The classical biological role of miRNAs is to degrade or to inhibit the translation of target genes to reduce the level of protein-coding genes expression [50]. In addition to a small number of verified cmiRNA–Cgene, the remaining were only predicted by the databases to have a targeted regulatory relationship. Although the relative expression levels between tumor and normal tissues and Spearman correlation analysis results preliminarily showed that there was a significant negative correlation between the expression of some miRNAs and predicted target genes, they require further experimental verification in the future. It is worth noting that miRNA can function outside classical paradigms such as coding for peptides, directly activating transcription, up-regulating protein expression, and activating toll-like receptors [51]. Overall, the constructed cmiRNA–Cgene network, GO enrichment analysis and GSEA showed potentially important cmiRNA–Cgene regulation pathways involved in the progression of LUAD, which should be the focus of future in-depth research.

Several shortcomings are present in this study: First, the cmiRNA was predicted by the database based on circadian genes. Although we set strict criteria to increase the reliability of this targeting regulation, and the partial targeting regulation relationship has been validated by other studies, the true targeting regulation relationship of them requires further verification. Second, owing to the difficulty in collecting clinical samples and the limited time, no large-scale clinical cohort study was carried out to verify the prediction model. Finally, the cmiRNA–Cgene network pathway and its target regulation mechanism were only preliminary explorations and require further in-depth validation.

## 5. Conclusions

To the best of our knowledge, as this is the first study to explore the role of cmiRNAs in lung adenocarcinoma. The established prognostic model based on cmiRNA pairs exhibited good performance in predicting the overall, progression-free survival and sensitivity to chemotherapy and targeted therapy.

## Figures and Tables

**Table 1:** Clinicopathological features of the patients.

Characteristics	TCGA–LUAD Cohort				GEO Cohort	WHUH Cohort
	Entire N = 450	Training Dataset N = 225	Testing Dataset N = 225	*p*-Value ^#^	GSE63805 N = 32	N = 11
**Age (years)**	65.2 ± 9.9	66.1 ± 9.4	64.3 ± 10.3	0.049	65.4 ± 11.9	57.6 ± 7.4
**Sex (%)**				0.257		
Male	212 (52.9)	112 (50.2)	100 (44.4)		15 (46.9)	8 (72.7)
Female	238 (47.1)	113 (49.8)	125 (55.6)		17 (53.1)	3 (27.3)
**Smoking Status**				1.0		
Yes	388 (86.2)	194 (86.2)	194 (86.2)		27 (84.4)	6 (54.5)
No	62 (13.8)	31 (13.8)	31 (13.8)		4 (12.5)	5 (45.5)
**TNM Stage (%)**				0.144		
I	249 (55.3)	136 (60.4)	113 (50.2)		27 (84.4)	7 (63.6)
II	108 (24.0)	46 (20.4)	62 (27.6)		4 (12.5)	4 (36.4)
III	74 (16.4)	33 (14.7)	41 (18.2)			
IV	19 (4.2)	10 (4.4)	9 (4.0)			
**T stage (%)**				0.713		
T1	156 (34.7)	76 (33.8)	80 (35.6)			8 (72.7)
T2	237 (52.7)	123 (54.7)	114 (50.7)			3 (27.3)
T3	42 (9.3)	18 (8.0)	24 (10.7)			
T4	15 (3.3)	8 (3.6)	7 (3.1)			
**N stage (%)**				0.055 ^f^		
N0	302 (67.1)	162 (72.0)	140 (62.2)			7 (63.6)
N1	82 (18.2)	31 (13.8)	51 (22.7)			4 (36.4)
N2	64 (14.2)	31 (13.8)	33 (14.7)			
N3	2 (0.4)	1 (0.4)	1 (0.4)			
**M stage (%)**				0.638 ^f^		
M0	297 (66.0)	145 (64.4)	152 (67.6)			11 (100)
M1	19 (4.2)	10 (4.4)	9 (4.0)			
Mx	132 (29.3)	68 (30.2)	64 (28.4)			
**OS time (days)**	891 ± 877	860 ± 763	921 ± 980	0.460	2173.5 ± 1230.5	
**OS state**				0.844		
Alive	290 (64.4)	146 (64.9)	144 (64.0)		27 (84.4)	
Dead	160 (35.6)	79 (35.1)	81 (36.0)		5 (15.6)	

**^#^** The difference between training and testing datasets was calculated using *t* test or chi-square test unless specified. ^f^: Fisher’s exact test. Means ± standard deviation (SD) was presented. OS: overall survival.

## Data Availability

Anonymized clinical data are available with completion of a data-sharing agreement and in accordance with the Wuhan Union hospital’s institutional review boards and institutional guidelines. Please submit requests for participant-related clinical and other data to Y.J. (whuhjy@126.com).

## Supplementary Materials

The following supporting information can be downloaded at: https://www.mdpi.com/article/10.3390/cancers14205106/s1. Table S1. The forward primer sequences of 11 cmiRNA in the prognostic model used in quantitative real-time polymerase chain reaction (qRT-PCR) analysis. Table S2. The list of circadian genes downloaded from the Circadian Gene Database (Supplied in a separate Excel file, Table S2). Table S3. The list of miRNAs in TCGA-LUAD and GSE63805 cohort and circadian miRNAs predicted by miRDB and miRDIP databases (Supplied in a separate Excel file, Table S3). Table S4. cmiRNA pairs associated with prognosis obtained by Univariate COX regression analysis (*p* < 0.01). Table S5. Corresponding cmiRNAs in the prognostic model in TCGA-LUAD and GSE63805 cohort. Table S6. Expression levels of miRNA of the model and its targeted genes in tumor tissue (compared with normal tissues) in TCGA-LUAD cohort. Table S7. cmiRNAs of the prognostic model and corresponding target Cgenes (Supplied in a separate Excel file, Table S7). Figure S1. Analysis of immune cells between the high- and low-risk group. Figure S2. Gene Set Enrichment Analysis (GSEA) between the high- and low-risk group.
