# Supplementary material for: Exploration of a Novel Circadian miRNA Pair Signature for Predicting Prognosis of Lung Adenocarcinoma
Source: Cancers (Basel). 2022 Oct 18;14(20):5106. doi: 10.3390/cancers14205106 (PMC9600995; doi:10.3390/cancers14205106)
Supplement: Supplementary file 1 [file cancers-14-05106-s001.zip › Supplementary materials.pdf]

Exploration of a Novel Circadian miRNA Pair Signature for Predicting Prognosis of Lung Adenocarcinoma

Zhengrong Yin[1,2]†, Jingjing Deng[1,2]†, Mei Zhou[1,2], Minglei Li[1,2], E Zhou[1,2], Jiatong Liu[1,2], Zhe Jia[1,2], Guanghai Yang[3*], Yang Jin[1,2*]

[1]Department of Respiratory and Critical Care Medicine, Hubei Province Clinical Research Center for Major Respiratory Diseases, NHC Key Laboratory of Pulmonary Diseases, Union Hospital, Tongji Medical College, Huazhong University of Science and Technology, Wuhan 430022, China.

[2]Hubei Province Engineering Research Center for Tumor-Targeted Biochemotherapy, MOE Key Laboratory of Biological Targeted Therapy, Union Hospital, Tongji Medical College, Huazhong University of Science and Technology, Wuhan 430022, China.

[3]Department of Thoracic Surgery, Union Hospital, Tongji Medical College, Huazhong University of Science and Technology, Wuhan 430022, China.

Supplementary materials

Table S1. The forward primer sequences of 11 cmiRNA in the prognostic model used in quantitative real-time polymerase chain reaction (qRT-PCR) analysis.

Table S2. The list of circadian genes downloaded from the Circadian Gene Database (Supplied in a separate Excel file, Table S2).

Table S3. The list of miRNAs in TCGA-LUAD and GSE63805 cohort and circadian miRNAs predicted by miRDB and miRDIP databases (Supplied in a separate Excel file, Table S3).

Table S4. cmiRNA pairs associated with prognosis obtained by Univariate COX

regression analysis (p<0.01).

Table S5. Corresponding cmiRNAs in the prognostic model in TCGA-LUAD and GSE63805 cohort.

Table S6. Expression levels of miRNA of the model and its targeted genes in tumor tissue (compared with normal tissues) in TCGA-LUAD cohort.

Table S7. cmiRNAs of the prognostic model and corresponding target Cgenes (Supplied in a separate Excel file, Table S7).

Figure S1. Analysis of immune cells between the high- and low-risk group.

Figure S2. Gene Set Enrichment Analysis (GSEA) between the high- and low-risk group.

Table S1. The forward primer sequences of 11 cmiRNA in the prognostic model used in quantitative real-time polymerase chain reaction (qRT-PCR) analysis.

miRNA	forward primer sequences
hsa-miR-584-5p	AAGTTGCATTATGGTTTGCCTGG
hsa-miR-539-3p	CCTGCTGGATCATACAAGGACAA
hsa-miR-382-5p	AACGGCGAAGTTGTTCGTGG
hsa-miR-376a-3p	GCGCATCATAGAGGAAAATCCACGT
hsa-miR-335-5p	ATGCGCTCAAGAGCAATAACGA
hsa-miR-31-5p	AACACGCAGGCAAGATGCTG
hsa-miR-215-5p	GCGGCCTGATGACCTATGAATT
hsa-miR-153-3p	AGCTGGACTTGCATAGTCACAAA
hsa-miR-138-5p	AATTGCTCAGCTGGTGTTGTGA
hsa-miR-136-5p	AAGCGCCTACTCCATTTGTTTTG
hsa-miR-133b	ACCGTCTTTGGTCCCCTTCAA

Table S4. cmiRNA pairs associated with prognosis obtained by Univariate COX regression analysis (p<0.01).

id	HR	HR.95L	HR.95H	*p* value
hsa-miR-1301-3p|hsa-miR-31-5p	0.413999396	0.259152073	0.66137036	0.000224451
hsa-miR-590-5p|hsa-miR-31-5p	0.403672516	0.252108247	0.646355296	0.000158758
hsa-miR-628-5p|hsa-miR-31-5p	0.401853234	0.250326331	0.645102019	0.000159942
hsa-miR-503-5p|hsa-miR-31-5p	0.498869725	0.317389351	0.784118946	0.002578261
hsa-miR-139-5p|hsa-miR-584-5p	0.455970827	0.288842241	0.719802597	0.000747842
hsa-miR-539-3p|hsa-miR-376a-3p	2.047587084	1.299731036	3.225754215	0.00199842
hsa-miR-153-3p|hsa-miR-133b	2.044139653	1.222537035	3.417898028	0.006409482
hsa-miR-584-5p|hsa-miR-335-5p	2.269786156	1.248127997	4.127725047	0.00722347
hsa-miR-409-5p|hsa-miR-31-5p	0.510762588	0.326515364	0.798977476	0.00324996
hsa-miR-409-3p|hsa-miR-31-5p	0.435636034	0.277673898	0.683459107	0.000298863
hsa-miR-187-3p|hsa-miR-31-5p	0.423664851	0.266291945	0.674041815	0.000289022
hsa-miR-187-3p|hsa-miR-215-5p	0.51737803	0.331035031	0.808615405	0.003823657
hsa-miR-508-3p|hsa-miR-31-5p	0.481034758	0.308091077	0.751058553	0.001285116
hsa-miR-432-5p|hsa-miR-31-5p	0.523238595	0.333715601	0.820395051	0.00476216
hsa-miR-136-5p|hsa-miR-31-5p	0.425624582	0.266537943	0.679664152	0.000347522
hsa-miR-136-5p|hsa-miR-382-5p	0.467632002	0.294520979	0.742492743	0.001272174
hsa-miR-138-5p|hsa-miR-31-5p	0.339818315	0.210702981	0.548053409	9.60E-06
hsa-miR-196b-5p|hsa-miR-151a-5p	1.853672806	1.162394144	2.956056592	0.009543416
hsa-miR-33a-5p|hsa-miR-31-5p	0.415053516	0.252430082	0.682444104	0.000528495
hsa-miR-335-5p|hsa-miR-31-5p	0.345515885	0.218742488	0.545761493	5.21E-06
hsa-miR-335-5p|hsa-miR-215-5p	0.472672695	0.284548304	0.785172407	0.003803736
hsa-miR-758-3p|hsa-miR-31-5p	0.463314163	0.29628723	0.724499714	0.000744079
hsa-miR-31-5p|hsa-miR-93-3p	2.714016346	1.704354779	4.321802491	2.60E-05
hsa-miR-31-5p|hsa-miR-382-5p	2.405022745	1.528351537	3.784557587	0.000148371
hsa-miR-31-5p|hsa-miR-326	1.995031156	1.251141308	3.181214852	0.003718432
hsa-miR-31-5p|hsa-miR-133a-3p	2.086912141	1.336857297	3.257791461	0.001205333

The cmiRAN pairs finally included in the model are marked in red.

Table S5. Corresponding miRNAs in the prognostic model in TCGA-LUAD and GEO cohort.

TCGA-LUAD cohort	GSE63805 cohort
hsa-miR-539-3p	hsa-miR-539
hsa-miR-153-3p	hsa-miR-153
hsa-miR-584-5p	hsa-miR-584
hsa-miR-136-5p	hsa-miR-136
hsa-miR-138-5p	hsa-miR-138
hsa-miR-335-5p	hsa-miR-335
hsa-miR-376a-3p	hsa-miR-376a
hsa-miR-133b	hsa-miR-133b
hsa-miR-382-5p	hsa-miR-382
hsa-miR-31-5p	hsa-miR-31
hsa-miR-215-5p	hsa-miR-215

Table S6. Expression levels of miRNA of the model and its targeted genes in tumor tissue (compared with normal tissues) in TCGA-LUAD cohort.

ID	LogFC	*p* value	ID	LogFC	*p* value
hsa-miR-539-3p	1.44	4.52E-04	NINJ1	0.26	2.99E-03
hsa-miR-153-3p	1.30	4.38E-06	CCDC85C	-0.11	5.66E-04
hsa-miR-584-5p	-1.11	2.87E-09	KDSR	-0.36	3.00E-15
hsa-miR-136-5p	2.32	1.73E-16	DDIT4	1.23	7.51E-09
hsa-miR-138-5p	-1.12	6.44E-07	OTUD4	-0.13	7.15E-04
hsa-miR-335-5p	-1.04	3.13E-13	RPL22	0.17	1.07E-02
hsa-miR-376a-3p	1.61	7.85E-05	INHBB	1.21	2.40E-04
hsa-miR-133b	-2.39	5.36E-31	QKI	-1.17	3.03E-30
hsa-miR-382-5p	1.32	1.34E-07	ZNF704	-0.65	8.09E-13
hsa-miR-31-5p	4.91	5.15E-18	GAB1	-1.16	3.80E-27
hsa-miR-215-5p	3.06	2.23E-09	ZBTB34	-0.12	4.76E-05
LHFPL6	-1.55	7.42E-31	RHOBTB1	-0.18	8.06E-06
TAGLN2	0.34	5.10E-04	FAM53B	-0.61	2.08E-14
UBA2	0.56	1.02E-09	LATS2	-1.05	8.34E-26
DUSP1	-1.46	8.44E-22	VEZT	0.55	1.33E-13
KIF3C	1.23	5.56E-08	BACH2	-0.52	1.81E-07
YES1	0.27	5.71E-02	SEPHS1	0.32	1.48E-06
RFFL	0.57	3.07E-11	FBXO28	0.63	5.24E-18
ELAVL1	0.36	1.21E-17	KDM4C	-0.03	2.67E-03
SHISA5	0.17	5.33E-03	BRWD1	0.51	4.47E-03
RABGAP1L	-0.25	1.36E-06	CRISPLD2	-0.49	1.18E-02
DOCK5	0.57	5.10E-02	XPR1	2.05	2.00E-30
RMND5A	-0.08	1.64E-03	PLAG1	0.38	7.79E-01
RARA	-0.48	3.73E-11	TOP1	0.50	7.97E-12
KLF11	-0.57	4.81E-15	ATP1B1	1.40	8.89E-16
ROCK2	-0.04	2.13E-02	NR1D2	-0.11	1.15E-01
EID1	-0.54	7.35E-19	CD200	-0.44	9.98E-05
GNG2	-0.71	5.05E-14	PPIL1	1.17	7.67E-26
SEH1L	0.52	2.38E-11	SH3PXD2A	0.47	7.15E-06
NSFL1C	0.29	2.53E-06			

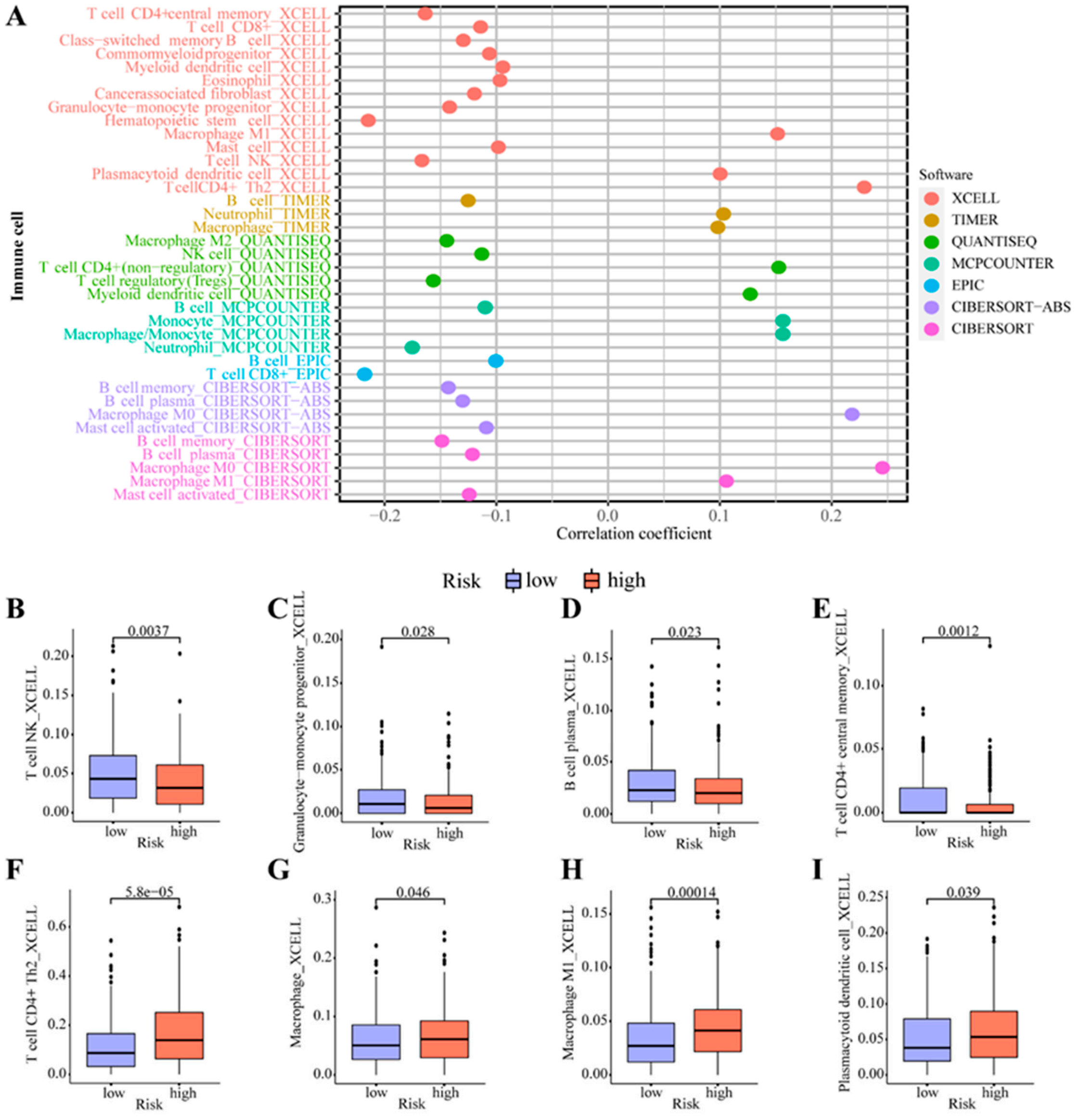

Figure S1. Analysis of immune cells between the high- and low-risk group

(A) Overview of significant correlation between risk score and immune cells by Spearman correlation analysis ($p < 0.05$). **(B-I)** Comparison of level of representative immune cells between the high- and low-risk group.

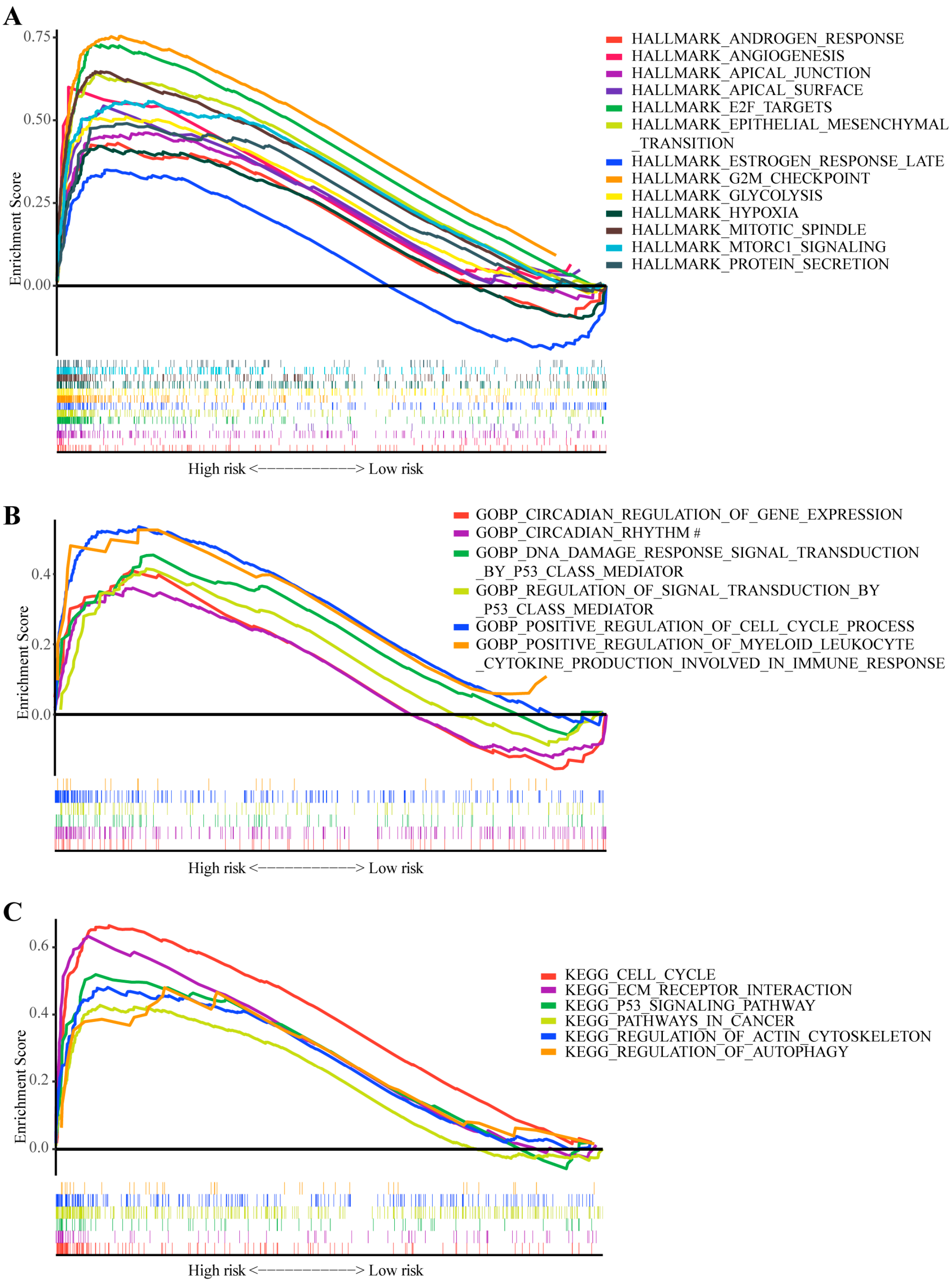

Figure S2. Gene Set Enrichment Analysis (GSEA) between the high- and low-risk group

GSEA plots depict the significant enrichment (FDR < 0.05) of **(A)** classical cancer hallmark gene sets and **(B, C)** other typical gene sets of tumor-related biological processes between the high- and low-risk group. (# FDR = 0.051).
